# Minimal invasive ostheosintesis for treatment of diaphiseal transverse humeral shaft fractures

**DOI:** 10.1590/1413-78522014220200698

**Published:** 2014

**Authors:** Rodrigo Kallás Zogaib, Steven Morgan, Paulo Santoro Belangero, Hélio Jorge Alvachian Fernandes, William Dias Belangero, Bruno Livani

**Affiliations:** 1 Universidade de Campinas, Hospital das Clinicas, Department of Orthopedics and Traumatology, Campinas, SP, Brazil, Department of Orthopedics and Traumatology, Hospital das Clinicas, Universidade de Campinas (Unicamp), Campinas, SP, Brazil; 2 Swedish Medical Center, Englewood, Colorado, USA, Mountain Orthopaedic Trauma Surgeons (MOTUS), Swedish Medical Center, Englewood, Colorado, USA; 3 Universidade Federal de São Paulo, Departament of Orthopedics and Traumatology, São Paulo, SP, Brazil, Departament of Orthopedics and Traumatology, Universidade Federal de São Paulo (UNIFESP) São Paulo, SP, Brazil

**Keywords:** Fracture fixation, internal, Surgical procedures, operative, Arm, Upper extremity

## Abstract

**OBJECTIVE:**

To evaluate patients with transverse fractures of the shaft of the humerus treated with indirect reduction and internal fixation with plate and screws through minimally invasive technique.

**METHODS:**

Inclusion criteria were adult patients with transverse diaphyseal fractures of the humerus closed, isolated or not occurring within 15 days of the initial trauma. Exclusion criteria were patients with compound fractures.

**RESULTS:**

In two patients, proximal screw loosening occurred, however, the fractures consolidated in the same mean time as the rest of the series. Consolidation with up to 5 degrees of varus occurred in five cases and extension deficit was observed in the patient with olecranon fracture treated with tension band, which was not considered as a complication. There was no recurrence of infection or iatrogenic radial nerve injury.

**CONCLUSION:**

It can be concluded that minimally invasive osteosynthesis with bridge plate can be considered a safe and effective option for the treatment of transverse fractures of the humeral shaft.*** Level of Evidence III, Therapeutic Study.***

## INTRODUCTION

From the years 1980, the development of minimally invasive techniques began to draw interest in Brazil for treatment of diaphysis fractures of long bones, especially the femur.^1^The concept currently known as minimally invasive osteosynthesis with plates (MIOP) is based on the relative stability of the fracture with minimal damage to the surrounding soft tissues. Relative stability of the fracture secondary promotes healing, and subsequent formation of the bone callus, and reduces the possibility of infection and non-union.^2^ ^-^ ^7^

MIOP techniques were initially recommended for the treatment of comminuted fractures, because they promote a biological fixation without devitalization of bone fragments.^2^ ^-^ ^7^ In the past 10 years, these principles have been widely used in the treatment of fractures of the humeral shaft, with good results.^8^ ^-^ ^15^

The MIOP technique is best used for the treatment of fractures with low strain.^2^ ^-^ ^4^ ^,^ ^7^

In the humerus, this technique has brought good clinical results, even in simple fractures traits. The aim of this study was to report the results of application of the minimally invasive technique with bridging plates in the treatment of transverse fractures of the humeral shaft analyzing the time of consolidation and the function.

## PATIENTS AND METHODS

Between November 2000 and April 2011, adult patients with transverse fractures of the humeral shaft underwent reduction and fixation using the MIOP technique up to 15 days after the initial trauma. Exclusion criteria were compound fractures, pathological fractures, time over 15 days of the initial trauma, associated neurovascular injury, and the presence of open growth plate. The inclusion criterion was the presence of transverse diaphysis fracture of the humerus treated by the technique MIOP.

All fractures were operated with the same technique described below, and rehabilitation was performed following the same protocol.

The follow-up period ranged from 6 to 126 months (mean 51.6 months). DASH score was used in the evaluation of all patients during postoperative follow-up period.^15^

### Surgical technique

The patient is kept in supine position on a standard operating table, and the arm to be operated is carefully leaned on a side support desk. Two assistants are needed during surgery; one assistant maintains traction with the semi-flexed elbow, and the other directly assists in the procedure, working in the surgical field.

Large fragments of narrow dynamic compression plates (DCP) with 12-hole are generally used. In the diaphyseal region, the plates need not to be molded because the anterior surface of the humerus is flat and fits perfectly to the implant. Two previous accesses are performed , one proximal and one distal with about 3 to 5 cm each, based on the original description and Thompson cited Henry Hoppenfeld and De Boer^16^, according to widely known techniques of surgical access. The distal access is usually accomplished first, between the biceps muscle and the brachial muscle. After visualization of the lateral cutaneous nerve of the forearm, the brachial muscle is sectioned longitudinally between its lateral third and medial two thirds, in a distance of about 3-5 cm to expose the anterior aspect of the humeral shaft. Hohmans' levers type retractors are avoided at this point to prevent compression, even indirectly, on the radial nerve.

The proximal access is performed between the tendon of the biceps muscle medially and the tendon of the deltoid muscle and the cephalic vein laterally. Then, the arm is abducted between 60 and 90 degrees to correct the typical varus deviation. The plate is inserted from proximal to distal. Traction is gently applied to restore the arm's length and fracture reduction. The distal fragment is rotated so that the axis between the long head biceps and the bicondylar axis is in a plane orthogonal so as to correct any rotational deviations. After indirect fracture reduction and proper seating of the plate, a loose proximal screw and one distal screw are inserted.

The final tightening of the distal screw and a radiographic control or image intensifier are performed. Rotational and angular deviations, and diastasis at the fracture are avoided, as long as possible, with a contact between the fragments of at least 50 percent in both AP and profile incidences. Two more proximal and distal screws in alternate holes are introduced for definitive fixation.

The incisions are sutured in a conventional manner with simple separated stitches. Drains or postoperative immobilization are not used.

After surgery, the patient is instructed to move the shoulder and elbow and use the newly operated arm for routine activities of daily life, such as eating and personal hygiene. Reassessment is done weekly for the first two weeks and monthly thereafter. All visits involve radiographs and clinical examinations to evaluate consolidation and functional rehabilitation of the patient, especially regarding the degree of mobility and function of the shoulder and elbow. ^8^ ^,^ ^17^

## RESULTS

**Figure 1:**
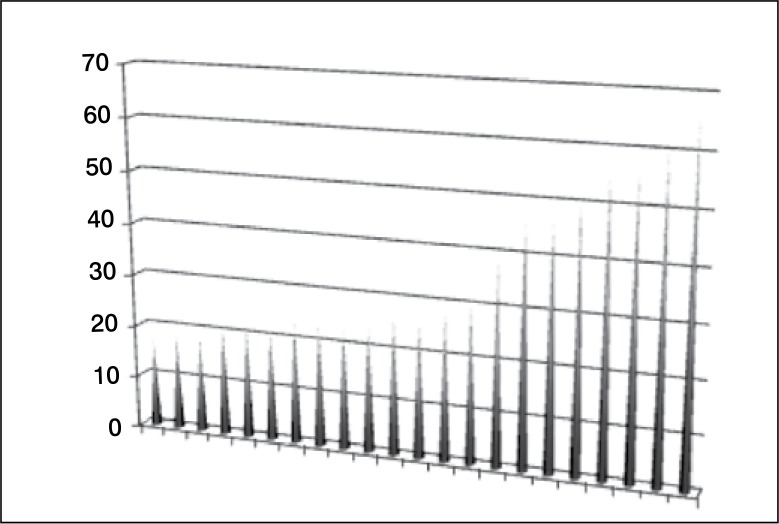
Age distribution on the group of studied subjects.

**Figure 2:**
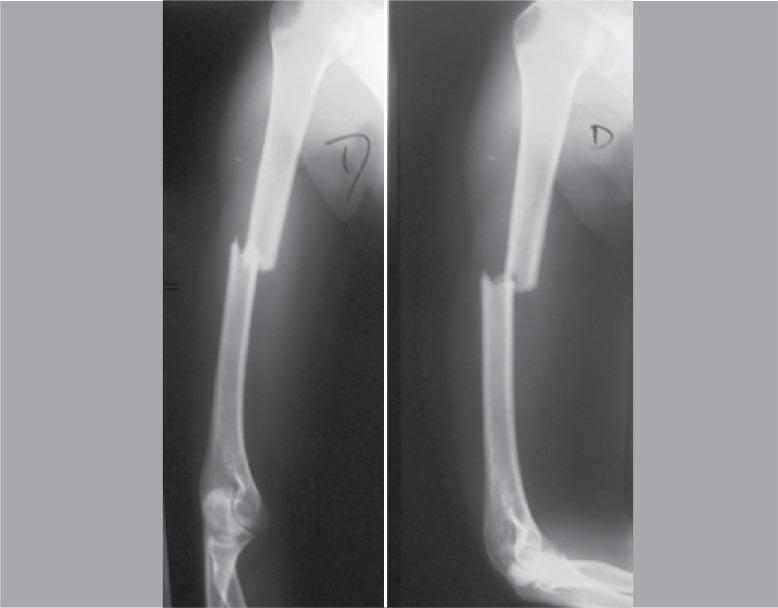
Preoperative anteroposterior and lateral radiographs incidence.

**Figure 3:**
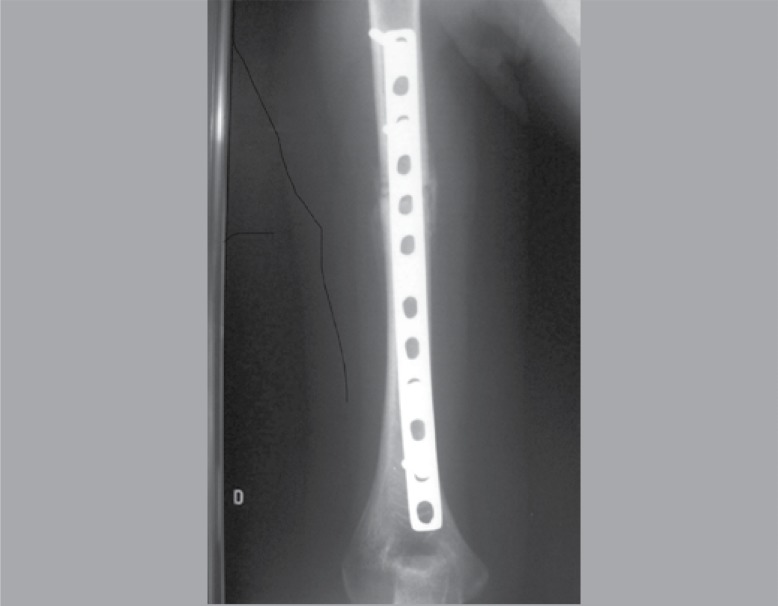
Anteroposterior incidence, 6 weeks after surgery.

**Figure 4:**
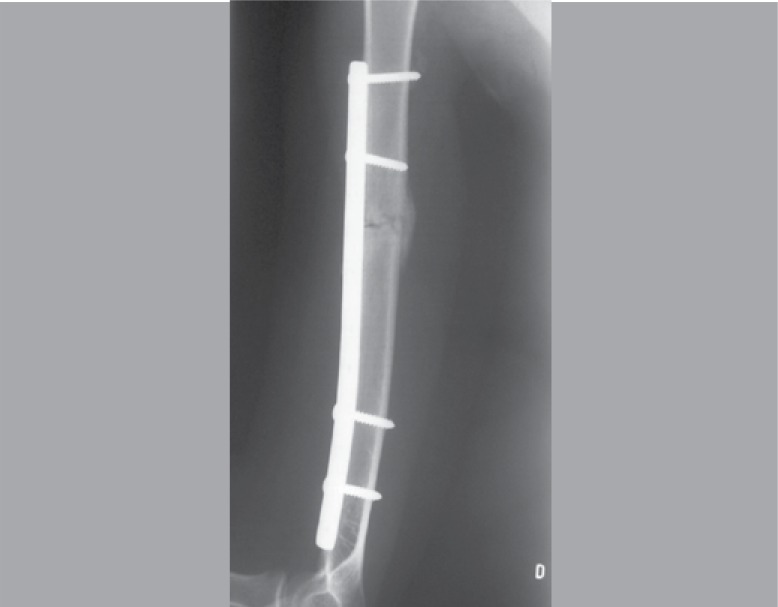
Lateral incidence six weeks after surgery.

**Figure 5:**
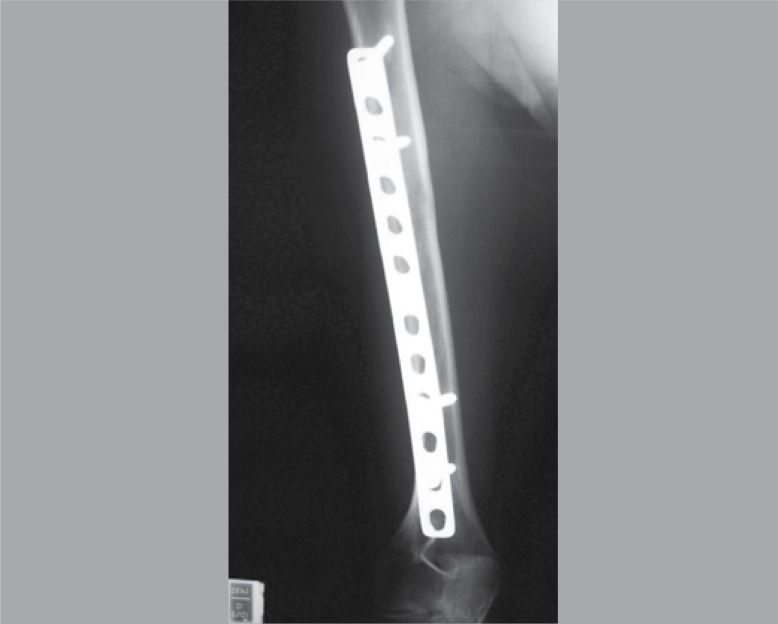
Anteroposterior incidence, 30 months after surgery.

Twenty two patients with 23 fractures were analyzed, being fifteen males (65%) and seven females (35%). The age of patients ranged from 18 to 66 years old (mean age 33.5 years old) years. (Figure 1) One of the patients in the series had bilateral fracture of the humeral shaft. All fractures consolidated and there was no neurovascular injury caused by the procedure. The fractures have consolidated within three months (mean 2.7 months). (Figures 2-5) The range of motion of the shoulder and elbow were symmetrical when compared to the uninvolved contralateral. (Figure 7) There were two complications both related to implant failure, more specifically of the proximal screws, in both cases, but even then there was fracture union in both cases. 

**Figure 6:**
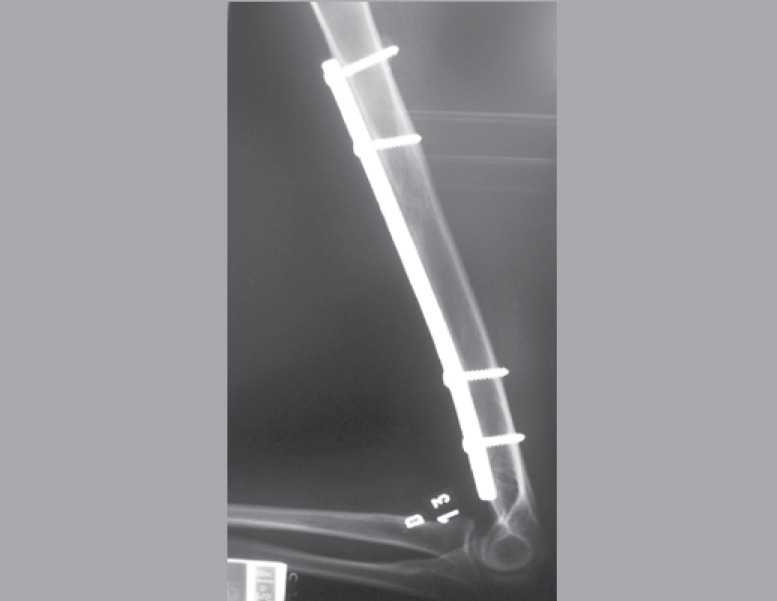
Lateral view, 30 months after surgery.

**Figure 7:**
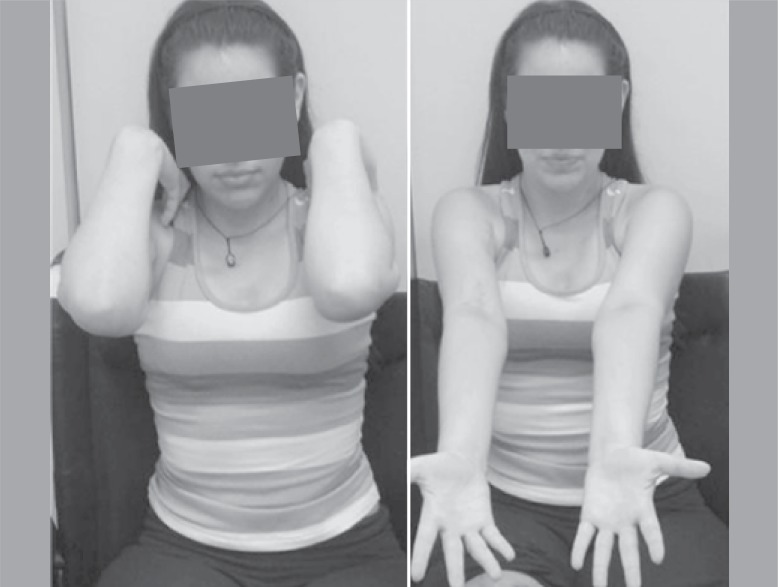
Thirty months after surgery, showing excellent arch of movement (fractured side: right).

One patient, who had a deficit of elbow extension had a ipsilateral olecranon fracture that was treated with open reduction and fixation with tension band, reason why the deficit was not rated as a complication, and five other patients had mild cubitus varus up to 5 degrees without clinical, aesthetic, or functional effects, which were also not considered as complications. There was no infection or iatrogenic radial nerve injury. DASH score ranged from 0 to 12.5 (mean 5.45). (Table 1)

**Table 1:** Patients' pre and post-operative data.

Age (years)	Gender	Side	Trauma mechanism	Associated injuries	Consolidation time (months)	Time of follow up (months)	Complications	DASH *score*
23	M	R	motorcycle accident	N	2	126	*cubitus varus*	10.8
66	M	L	fall	N	2	125	N	2.5
57	M	L	fall	N	2	125	N	3.3
17	F	R	running over	N	3	124	N	0
48	M	L	automobile accident	forearm fracture	2	123	*cubitus varus*	18.3
50	M	L	motorcycle accident	N	3	44	N	1,7
24	M	R	motorcycle accident	N	3	44	N	0
18	F	R	motorcycle accident	right clavicle fracture	2	44	N	0
21	F	R	motorcycle accident	forearm fracture lllc + sd compartimental	3	42	N	22.5
26	M	L	motorcycle accident	Left tibia + patella + femur fracture	2	39	N	0.8
28	M	L	motorcycle accident	N	3	36	Proximal screw breakage	1.7
38	M	L	motorcycle accident	N	3	35	*cubitus varus*	2.5
18	M	L	motorcycle accident	Acute subdural bleeding	3	35	N	19.2
21	F	L	automobile accident	contralateral humerus fracture	3	32	N	0
21	F	R	automobile accident	contralateral humerus fracture	3	32	N	0
60	M	L	fall	N	2	30	Proximal screw breakage	5.0
23	M	L	motorcycle accident	left olecranon fracture	3	30	cubitus varus	15.8
17	F	R	motorcycle accident	Femur fracture and right llla (*) tibia	2	30	n	0
47	M	L	motorcycle accident	N	2	30	*cubitus varus*	0
30	M	L	fall	Left ankle fracture	3	18	N	0
26	M	L	running over	Left tibia fracture	2	10	N	0
23	F	R	motorcycle accident	Left femur fracture	3	8	N	0
56	F	L	automobile accident	left distal femur fracture head trauma	3	6	N	15.8

## DISCUSSION

**Figure 8:**
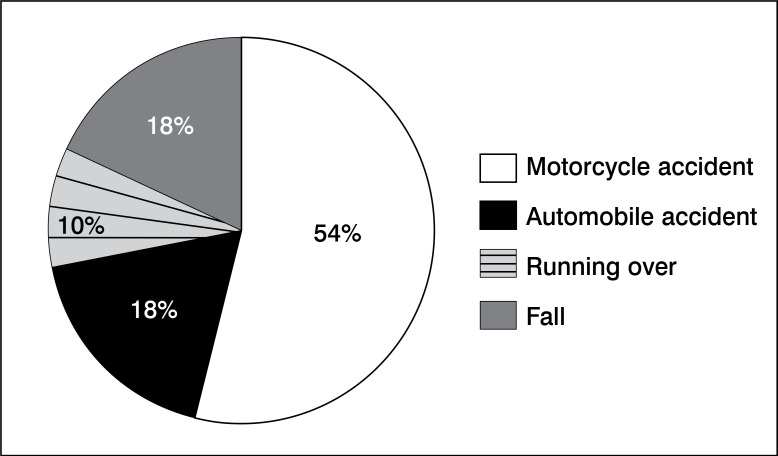
Ethiologic distribution of the group of studied subjects.

The incidence of fractures of the humeral shaft in North America is 20 per 100,000 people per year. Half of these cases involve the middle third of the humeral shaft, and 20 to 30 % involve the distal third. Transverse fractures are more common in the middle third, and oblique lines and spirals in the distal third of the humeral shaft. Over the past 20 years, a higher incidence of comminuted fractures of the distal third of the shaft by the increase of injuries caused by high energy trauma has been observed.^11^(Figure 8) Conservative treatment of fractures of the humeral shaft in isolation can achieve good results; angular and rotational deviations are well compensated by the large range of motion of the shoulder with little or no aesthetic or functional impairment.^8^ ^,^ ^17^

Surgical treatment is necessary in cases of multiple trauma, multiple fractures or compound fractures, bilateral fractures, floating elbow, loss of reduction or unacceptable reduction and when there is need for surgical exploration of the radial nerve.^8^ ^,^ ^12^ ^-^ ^14^ ^,^ ^17^ ^-^ ^19^ Of 22 cases reported in this paper, 13 presented with bilateral fracture of humerus, fracture of the ipsilateral upper limb, multiple fractures or were polytraumatized patients. Of the remaining patients (nine cases), MIOP indication was made because of failure in achieving an acceptable reduction or keeping it.

Transverse fractures of the humerus are considered relative indications to advice for surgical treatment, due to the difficulty of maintaining an acceptable reduction, and the possibility of delayed union, non-union and pseudarthrosis. Surgical options include locked intramedullary nail and conventional osteosynthesis with plate.^4^ ^,^ ^13^ ^,^ ^17^ ^,^ ^20^ The intramedullary nails has been less promising in the humerus than in the lower limb. Osteosynthesis with conventional plate present considerable surgical morbidity and risk of non-union, infection, and iatrogenic radial nerve injury.^8^ ^,^ ^11^ ^-^ ^14^ ^,^ ^17^In this study, all fractures consolidated and no neurological injury or infection were observed.

The aim of this study was not to suggest a single type of surgical treatment for all transverse fractures of the humeral shaft. In our opinion, surgical treatment of isolated diaphysis fractures of the humerus should be the exception and not the rule. However, in the presence of surgical indication, the MIOP technique should be included among the therapeutic options. Many authors have reported satisfactory results by applying the MIOP technique in the treatment of diaphysis fractures of the umerus.^8^ ^-^ ^14^ ^,^ ^17^ ^,^ ^21^ Simple tract fractures, however, show a high degree of relative deformation between bone fragments (strain), and theoretically should not be treated with the relative stability methods, and would not stand up to the mechanical demands until consolidation of the fracture.^2^ ^-^ ^10^ Nevertheless, the humerus has a high biological potential, that leads to a rapid consolidation. If soft tissues are preserved through a low trauma biological surgical technique, proper contact between fragments, and relative stability with elastic synthesis, even simple fractures should be treated by the MIOP technique because the mechanical demand is much lower in the upper limb when compared to the lower limb. Thus, the fracture consolidates before implant brings on fatigue. There are two critical factors for successful treatment with MIOP in transverse fractures of the humeral shaft: careful manipulation of the soft parts, and the quality of intraoperative reduction. Although the humerus has a high tolerance for angular or rotational deviations, diastasis of the fracture and translational deviation with possible insertion of soft tissue should be definitely avoided. The excellent success rates shown here can be reproduced and justified by the quality of intraoperative reduction. In all cases, the goal was to achieve at least 50% of bone contact in the anteroposterior plane and a maximum of 10 degrees of angular or rotational deviation. Despite efforts by the surgeon in these cases, five patients presented with slight varus, but did not compromise the final result, but could be avoided. According to Hunsaker *et al.,* ^22^ DASH score shown in this series was similar to that of the normal population.

Two patients had fatigue of synthesis material (loosening of the proximal screws), when they returned to their work activities, before complete fracture healing in the first 30 days after surgery. Both patients reported excessive physical effort and raised more than 30 Kg in the first month postoperatively. 

In both cases, the fractures consolidated without further problems.

The results presented in this paper are consistent with those reported by other authors who used different methods already consolidated in literature.^8^ ^-^ ^14^ ^,^ ^20^

## CONCLUSION

Osteosynthesis with bridge plate with minimally invasive technique can be considered an attractive alternative for the treatment of transverse fractures of the humeral shaft. Its use proved to be easy, safe and effective, respecting the technique described.
